# The Impact of Flavonoids and Omega-3 in Mitigating Frailty Syndrome to Improve Treatment Outcomes in Peripheral Artery Disease (PAD) Patients

**DOI:** 10.3390/nu17142303

**Published:** 2025-07-12

**Authors:** Sanaz Jamshidi, Zahra Eskandari, Amirhossein Faghih Ojaroodi, Shayan Keramat, Agata Stanek

**Affiliations:** 1 Department of Hematology, Faculty of Medical Sciences, Tarbiat Modares University, Tehran 1411713116, Iran; s19n93z@gmail.com; 2 Department of Hematology, Faculty of Allied Medicine, Bushehr University of Medical Sciences, Bushehr 7514633341, Iran; zhraaeskndrii@gmail.com; 3 Student Research Committee, Tabriz University of Medical Sciences, Tabriz 5165665931, Iran; amirfaghih1103@gmail.com; 4 VAS-European Independent Foundation in Angiology/Vascular Medicine, Via GB Grassi 74, 20157 Milan, Italy; shayan.sk1993@gmail.com; 5 Support Association of Patients of Buerger’s Disease, Buerger’s Disease NGO, Mashhad 9183785195, Iran; 6 Department of Internal Medicine, Metabolic Diseases and Angiology, Faculty of Health Sciences in Katowice, Medical University of Silesia, Ziołowa 45/47, 40-635 Katowice, Poland

**Keywords:** peripheral artery disease, frailty syndrome, Mediterranean diet, flavonoids, omega-3 fatty acids, inflammation, oxidative stress

## Abstract

Peripheral artery disease (PAD) is a common vascular disorder in the elderly, often accompanied by frailty syndrome, which is associated with increased inflammation, oxidative stress, and functional decline. Nutritional strategies, particularly those involving bioactive compounds like flavonoids and omega-3 fatty acids, have been suggested as potential approaches to modulate these pathological processes. This narrative review summarizes current evidence regarding the anti-inflammatory and antioxidant effects of flavonoids and omega-3 fatty acids, and their possible roles in mitigating frailty syndrome in patients with PAD. We examine mechanistic pathways including NF-κB, AMPK, PI3K/Akt/mTOR, and Nrf2, which are implicated in chronic inflammation, endothelial dysfunction, and muscle wasting. Although studies in general and aging populations suggest beneficial effects of these compounds on vascular and muscle health, specific evidence in PAD patients remains limited. Flavonoids may reduce pro-inflammatory cytokine production and enhance antioxidant responses, while omega-3 fatty acids have shown potential in modulating inflammatory signaling and supporting vascular repair. Current data provide a basis for further investigation into the dietary modulation of frailty syndrome in PAD. Understanding the impact of these nutrients may offer insights into adjunctive strategies for improving patient outcomes.

## 1. Introduction

Peripheral artery disease (PAD), characterized by the build-up of fatty plaques that narrow or obstruct arteries, stands as a significant contributor to cardiovascular diseases, resulting in substantial disorders and mortality [1]. It strongly indicates a high mortality rate, and the reason specifically is due to cardiovascular issues. Research indicates that PAD elevates the risk of all-cause mortality by 60%, cardiovascular-related deaths by 96%, coronary artery disease development by 45%, and cerebrovascular disease development by 35% [2]. The true prevalence of PAD is challenging to ascertain due to its often-asymptomatic nature, but studies indicate that 8.5 to 12 million individuals in the United States live with PAD [3]. Global data from 2019 showed 56.4 million cases in the 40–70 age range, and 57.0 million cases in individuals over 70 [4]. Smoking, high blood pressure, diabetes mellitus, dyslipidemia, thyroid dysfunction, heart failure, unsuitable diet, high cholesterol intake, and high BMI are among the risk factors for PAD [5].

Frailty syndrome (FS) defined in recent decades is generally understood as an age-associated decline in the ability to withstand stressors, not a disease itself, but a condition that significantly increases the risk of developing acute and chronic illnesses and disabilities [6]. It is a complex condition stemming from multiple interconnected biological processes, including inflammation, hormonal imbalances in the hypothalamic–pituitary axis, and disruptions in anabolic and catabolic hormones [7,8]. FS is a significant challenge in cardiac rehabilitation, often preventing frail patients with coronary heart disease from receiving necessary procedures and rehabilitation programs. Given the increasing elderly population, frailty is becoming a critical social and medical concern. Having a healthy diet and exercise are considered the most effective therapy for managing frailty, enhancing quality of life, and promoting independence in older adults after frailty detection [9].

The Mediterranean diet (MedDiet) is globally recognized and extensively researched. The MedDiet historically correlated with lower rates of chronic diseases and higher life expectancy. The traditional MedDiet emphasizes plant-based foods with moderate intake of dairy products like cheese, kefir, and yogurt. Eggs, fish, and poultry are eaten in low to moderate amounts, while red meat consumption is low. So, this diet is full of olive oil, flavonoids (vegetables, fruits), and omega-3(fish) [10]. Studies and epidemiological evidence indicate that chemicals such as statins [11], vitamins D [12], omega-3 fatty acids [13], and dietary polyphenols [14] are associated with reduced risk and progression of PAD and related cardiovascular diseases (CVDs) by decreasing inflammation, oxidative stress, and atherosclerosis. These findings support their protective roles in vascular health.

Studies have indicated the benefits of MedDiet for various disease and specially for cardiovascular disease incidence [15,16].

In the present review, we aim to investigate the role of the Mediterranean diet and the omega-3 and flavonoids that it contains to improve the treatment of PAD patients directly or as a supplement.

## 2. Frailty Syndrome Definition, Prevalence, and Causes

Frailty syndrome (FS) is a condition of increased vulnerability caused by age-related declines in body functions, reducing the ability to handle stress [17].

It is often diagnosed using Fried’s model, which considers three or more out of five signs: weak hand strength, exhaustion, slow walking, limited activity, and unintentional weight loss. A pre-frail stage, with one or two signs, indicates a high risk of developing full FS [18].

The prevalence of FS worldwide varies depending on the assessment method and population studied, but it generally ranges from 12% to 24% [19].

In the U.S., among adults aged 65 and older, frailty prevalence ranges from 7% to 12%. Rates increase with age, from about 4% in ages 65–74 to 25% in those 85 and older. Women are more likely to be frail than men (8% vs. 5%) [18]. In Latin American and Caribbean cities, frailty among adults aged 60 and older ranges from 30% to 48% in women and 21% to 35% in men [20]. In a study of 2,488 people aged 65 and older, about 41.8% were pre-frail, and 8.4% frail. FS increased with age and was more common among those with disabilities, depression, hip fractures, and other health conditions, regardless of sex, education, or living area [21].

Oxidative stress (OS) has been implicated as a key factor in the development of frailty, with early research in the 1950s pointing to its role [22].

OS contributes to aging and diseases like cardiovascular, neurodegenerative, chronic obstructive pulmonary disease, and cancer in older adults, with OS damage arising from imbalances in reactive species [23]. Studies show elevated OS markers—such as serum albumin, high-sensitivity C reactive protein (hs-CRP), 8-hydroxydeoxyguanosine (8-OdG), glutathione disulfide (GSSG), malondialdehyde (MDA), and 4-hydroxynonenal (4-HNE)—in frail individuals, with levels increasing with FS severity [24,25]. OS is also linked to sarcopenia [26], an early stage of FS, marked by increased OS markers, lipid peroxidation, and inflammatory indicators like IL-6 and prostaglandins [27]. Additionally, research has shown that increased levels of interleukin-6, isoprostaglandin, and lipoprotein phosphorylation A2 correlate with a higher incidence of FS [28]; it suggests that frailty is associated with OS, and OS-related biomarkers in serum may serve as potential predictive markers for FS [29].

A multi-center observational study found that telomere length was shortened due to increased OS indicated by elevated superoxide dismutase (SOD) activity and reduced total antioxidant capacity against ROS [26]. However, a different study found only telomere length, and not lipid peroxidation, to be a significant contributor to the frailty phenotype [30].

Another study has demonstrated a correlation between FS and circulating inflammatory and OS markers. Specifically, levels of TNF-alpha, GSSG, MDA, and 4-hydroxy-2,3-nonenal-protein plasma adducts were significantly higher in frail elderly individuals compared to their non-frail counterparts [25]. It has also been reported that there is a positive correlation between total antioxidant capacity and FS, and also between IL-6 and FS, even after adjusting for age and body mass index. Furthermore, in patients with cerebral small vessel disease and cognitive frailty, elevated levels of MDA (indicating lipid peroxidation) and lower SOD activity were observed alongside increased inflammatory markers like CRP, IL-6, and TNF-α [31,32,33]. Also, another study in Poland revealed a relationship between inflammatory markers (IL-6 and CRP) and FS in elderly participants [34]. Additionally, Marcos-Pérez et al. established strong quantitative associations between CRP and IL-6 as inflammatory biomarkers in frail older adults [35], while Ribeiro and colleagues suggested that CRP may serve as a biomarker of age-related frailty [36]. Furthermore, a meta-analysis identified CRP, vitamin D, albumin, hemoglobin, and (in men) free testosterone as biomarkers associated with FS [37].

Abnormalities in leukocytes and inflammatory markers are linked to FS [38]. In postmenopausal women, elevated fibrin turnover and fibrinolysis markers independently predicted FS [39]. Moreover, a meta-analysis found that frail and pre-frail individuals had significantly higher levels of inflammatory markers like CRP, IL-6, white blood cells, and fibrinogen compared to robust individuals [40]. Additionally, increased pro-inflammatory cytokines such as IL-1β, IL-18, IL-8, and CXCL10 are associated with FS [41].

## 3. The Relationship Between Frailty Syndrome and Chronic Vascular Diseases

Atherosclerosis is a cause of stroke and cardiovascular disease (CVD), which is known as an inflammatory disease of the large arteries [42]. In most people with atherosclerosis, it is an inactive disease, and its multiple pathological changes cause the activation of immune cells in vulnerable areas of the artery and disrupt lipoprotein regulation throughout the patient’s life [43,44]. Important risk factors for atherosclerosis include lipoprotein metabolism [45], hypertension [46], diabetes [47], obesity and nutrition [48], exercise and physical activity [49], stress and sleep [49,50], smoking [51], pollution [41], gut microbiota [52], alcohol consumption [53], and infection [54].

FS is a common disorder associated with aging, with adverse outcomes including falls, hospitalization, and mortality. CVD, which is frequently seen in the elderly population, is a leading cause of death, and it has been shown to be an important risk factor for FS [55].

### 3.1. Peripheral Arterial Disease

One of the diseases that results from atherosclerosis is PAD. While this disease refers to the involvement of any artery outside the brain and heart, it most commonly affects the lower extremities. Chronic limb-threatening ischemia (CLTI) and intermittent claudication (IC) are forms of PAD [56]. Although the disease is more common in older adults living in high-income countries, PAD has recently become a global problem [57,58]. The incidence of PAD increased by 25% in 2010 compared to 2000, with 200 million people affected, with the increase being greater in low- and middle-income countries than in high-income countries, and the upward trend continued, with 235 million people affected by PAD by 2015 [59]. The prevalence of symptomatic PAD depends on the disease experienced in primary care. In high-income countries, although people under 50 years of age showed a prevalence of less than 1%, people over 65 years of age had a prevalence of 6% [60]. If we do not consider the global population, the prevalence of IC is higher in men than in women [61]. The increase in the prevalence of PAD with the increase in the elderly population is undeniable, as is the case for diabetes [62]. In addition, the decrease in mortality in patients with stroke and myocardial infarction also promises to increase the survival of more people with PAD [63].

### 3.2. Frailty Syndrome and PAD

FS increases the risk of hospital admission, mobility issues, falls, and even death [18]. Frail patients are more likely to develop serious illnesses, and its negative impact on older adults’ health is well established. In vascular diseases, frailty is also recognized as a risk factor for complications [64]. Cardiovascular events including stroke, PAD, and coronary artery disease occur more frequently in frail individuals compared to non-frail individuals, and the fertility Index is associated with coronary and cerebral atherosclerosis [65].

PAD is associated with increased adverse health outcomes and mortality as a result of frailty syndrome [66]. Comparison of gait between frail and non-frail participants shows that this measure is worse in frail individuals [66]. One of the measures that is effective in diagnosing PAD is the ankle-brachial index (ABI), which is obtained from the ratio of ankle systolic blood pressure to brachial artery pressure [67]. Foot ulcers, neuropathy, and arterial stiffness reduce the sensitivity of ABI in diagnosing PAD in diabetic patients [68].

It has been shown that the ABI value is lower in the frail and non-frail group compared to the frail group. The results show that the gait speed is worse in the frail group. This indicates that these patients are more prone to poor balance, strength, and mobility, all of which are related to the diagnostic criteria for frailty syndrome [69]. Also, in a study con-ducted by Fang et al., it was found that there is a significant increase in the Modified Frailty Index (mFI) in patients who underwent lower limb amputation, and it increases the risk of hospitalization [70]. In addition, the association between frailty and increased mortality in people who underwent amputation was published by Campbell et al. [71]. Their results given by Helm et al. indicate that amputation is effective in reducing the patient’s functional capacity and dependence and plays a significant role in the development of frailty syndrome [72]. The effectiveness of treatment, the patient’s clinical course, and the complications of chronic lower limb ischemia are dependent on frailty characteristics, and these complications and symptoms in PAD are more likely to occur when accompanied by FS [73]. Statistics also show that patients with PAD and FS are 2.11 times more likely to die than non-frail individuals [74]. Other studies have also confirmed that patients with FS and PAD suffer from greater disability and mortality, and, in particular,, the Groningen frailty indicator (GFI) and modified essential frailty toolset (mEFT) were among the measurement indicators that were of greater value [75]. In line with these studies, it has been concluded that FS is associated with arterial stiffness in elderly patients and that they share common risk factors and pathophysiological mechanisms [69,76].

In addition to causing poor balance and gait difficulties in PAD patients, FS has been shown to contribute significantly to reduced amputation-free survival and overall survival [77]. Diabetes was identified as a major risk factor for PAD in a systematic review, with PAD occurring at twice the prevalence in the diabetic population compared to non-diabetic patients [78]. Persistent hyperglycemia, inflammation, increased OS, and insulin resistance that occur in diabetic patients further contribute to FS in these patients [79]. For older patients, where both PAD and frailty are prevalent, we can delay this with nutritional interventions and exercise [80]. It is clear that quality of life is a well-known factor in the prevention of frailty, and patients require ongoing care after discharge. Family support, financial status, age, multiple medication use, disease awareness, comfort with treatment, and mobility will all be influential factors [81]. The symptoms such as non-healing wounds, pain, and necrosis may indicate the need for surgery in PAD patients. Preoperative assessment of FS in these patients can help guide postoperative interventions such as physiotherapy, medication, and nutrition [82].

### 3.3. Frailty and Treatment Outcomes in PAD

Endovascular, medicinal, and surgical procedures are intended to improve patients’ quality of life and lessen the effects of reduced blood supply to the lower limbs [80,81]. Recent literature reviews have suggested that both endovascular and surgical approaches can be efficacious for patients with PAD; however, determining the optimal timing for intervention and the criteria for patient selection continues to pose significant challenges [83]. Among individuals with PAD, particularly those undergoing revascularization, the prevalence of frailty ranges from 20% to 60%, highlighting its substantial impact on treatment outcomes [84].

FS has emerged as a significant independent risk factor for both morbidity and mortality following vascular surgical and endovascular interventions [85]. Some previous studies have shown the prevalence and prognostic implications of FS in patients undergoing vascular procedures [86]. Frail patients are more vulnerable to postoperative complications, with studies indicating a threefold increase in 30-day mortality and a twofold increase in overall mortality compared to non-frail counterparts [84].

Supporting these observations, Brahmbhatt et al. [87] analyzed outcomes in 24,645 patients undergoing lower limb revascularization (92% surgical, 8% endovascular) and identified both frailty (mFI > 0.25) and female sex as significant predictors of complications. Similarly, Gonzalez et al. [88] reported that FS independently predicted major amputation (HR = 2.16), mortality (HR = 2.62), and the combined outcome (HR = 1.97) in a cohort of 431 PAD patients; notably, FS was associated with a sixfold increased risk of limb loss in those receiving endovascular therapy (OR = 6.28). In another study, Rothenberg et al. [89] used the risk assessment index (RAI) to demonstrate a stepwise increase in 30-day mortality following suprainguinal and infrainguinal revascularization procedures, with rates reaching 13.9% and 9.4%, respectively, in very frail patients.

Moreover, a systematic review identified significant associations between frailty and postoperative dependence in activities of daily living (ADL) after vascular procedures [80]. FS has also been linked to a higher incidence of severe postoperative complications, including Clavien–Dindo class IV events, major adverse cardiovascular outcomes, graft or prosthesis failure, infections, poor functional recovery, and cognitive decline [75,90].

## 4. Mediterranean Diet and Its Effects on Frailty Syndrome and Chronic Vascular Diseases

The MedDiet, a term coined around 1960, stands out as a globally recognized and extensively researched dietary pattern [91].

The MedDiet is most closely associated with traditional olive-growing regions and has historically correlated with lower rates of chronic diseases and longer lifespans. However, recent shifts in dietary habits and lifestyles have somewhat obscured these connections [92]. The core characteristics of the traditional MedDiet involve a high consumption of plant-based foods (fruits, vegetables, minimally refined cereals like bread, potatoes, beans, nuts, and seeds), emphasizing minimally processed, seasonally fresh, and locally sourced ingredients [93].

Olive oil, especially virgin and extra-virgin, is the primary fat source. Dairy intake is moderate, mainly through cheese and yogurt [94]. The generous intake of nuts, olive oil, and moderate wine consumption, particularly red wine during meals, distinguishes the MedDiet from other healthy dietary approaches, though it remains primarily plant-based. Individual components of the MedDiet, such as extra-virgin olive oil and nuts, have well-established health benefits [94,95].

However, recent research emphasizes the potential synergistic or additive health benefits derived from the overall combination of foods within the dietary pattern. The most compelling and consistent evidence supports the beneficial effects of the MedDiet on cardiovascular risk factors and CVD incidence [15,16]. Moreover, a substantial amount of research suggests potential benefits for other health conditions, including type 2 diabetes (T2D), metabolic syndrome (MetS), obesity, cancer, cognitive decline, and CVD mortality. The MedDiet has been consistently linked to a reduced risk of CVD outcomes across different populations.

Numerous prospective cohort studies have investigated this association further. For instance, a large prospective study involving 74,886 women from the Nurses’ Health Study (NHS) over 20 years found that greater adherence to the MedDiet, as measured by a higher aMED score, was associated with a 29% reduced risk of coronary heart disease (CHD) incidence and a 13% decreased risk of stroke in women [96].

Subsequent research from the same group, incorporating male health professionals from the Health Professional Follow-up Study (HPFS), indicated that an increase in the aMED score from baseline to the initial 4 years of follow-up was linked to a 9% lower CVD risk during the subsequent 20 years, suggesting that improved adherence to diet quality scores over time correlates with a significantly lower CVD risk in both the short and long term [97]. Similar findings have been reported in European populations. The EPIC-Spain cohort study, for example, demonstrated that adherence to the MedDiet was associated with a 27% lower risk of CHD [98]. Furthermore, EPIC research revealed that a 2-point increase in a MedDiet score was associated with a 25% reduced risk of all-cause mortality in a Greek population [99] and an 8% lower risk of all-cause mortality in older adults from nine European countries [100].

More recently, another comprehensive systematic review and meta-analysis encompassing 38 cohorts revealed inverse associations between higher MedDiet adherence and CVD mortality, CHD incidence, CHD mortality, stroke incidence, stroke mortality, and myocardial infarction (MI) incidence, when comparing the highest versus the lowest categories of MedDiet adherence [16].

### 4.1. Role of Omega-3 (Fish) in CVD

Omega-3 polyunsaturated fatty acids, abundant in fish, are key components of the widely researched Mediterranean diet. The American Heart Association/American College of Cardiology recommends consuming fish twice weekly for individuals with existing CVD [101]. Numerous meta-analyses suggest that increased fish consumption may reduce CVD morbidity and mortality, particularly in secondary prevention [102,103,104,105,106,107,108].

A systematic review of randomized controlled trials (RCTs) indicates that marine omega-3s are associated with a reduction in CVD risk [109]. The positive impacts of fish on CVD are believed to be mediated by improvements in lipid profiles [110], reductions in blood pressure [111,112], and potentially through reduced inflammation, oxidation, and coagulation [113]. Consequently, consuming fish in moderation appears to offer CVD benefits, whether as a component of the Mediterranean diet or independently, making it a beneficial dietary choice for those seeking heart-healthy eating habits.

### 4.2. Role of Fruits and Vegetables (Contains Flavonoids) in CVD

Cardiovascular health guidelines from organizations like the European Society of Cardiology (ESC) [114] and American Heart Association (AHA) [115] consistently emphasize the importance of consuming multiple daily servings of fruits and vegetables. These groups specifically recommend fruits and vegetables to lower CVD risk and highlight the value of phytochemicals, abundant in these foods, for disease prevention [116]. Numerous observational studies suggest that increased fruit and vegetable consumption is associated with improvements in risk factors. For instance, a 2003 study reported a systolic blood pressure reduction in women who consumed more fruits, vegetables, or vitamin C [117]. Additionally, some studies also have found an inverse relationship between fruit and vegetable intake and blood pressure [118,119].

A meta-analysis involving approximately 200,000 participants indicated that each serving of vegetables was associated with a 4% relative risk reduction in CVD, while each additional serving of fruit daily was linked to a 7% reduction. However, these findings were somewhat affected by heterogeneity and publication bias [120]. Another extensive meta-analysis of observational studies demonstrated a 17% decrease in CVD events with a daily intake of three to five servings of fruits and vegetables [121,122]. More recently, the EPIC-Heart study revealed a 22% lower risk of fatal ischemic heart disease in individuals consuming eight portions of fruits and vegetables a day compared to those consuming three or less, after an eight-year follow-up [123]. A RCT did demonstrate a statistically significant effect of fruit and vegetable intake on plasma antioxidant concentrations and blood pressure [124].

The potential benefits of fruits and vegetables may be attributable to reduced overall calorie intake or the presence of various micronutrients [125]. While the antioxidant properties and the benefits of flavonols found in fruits and vegetables are well-documented, alternative mechanisms, such as the effects of nitric oxide or weight loss associated with fruit-and-vegetable-rich diets, may also contribute [126].

## 5. Discussion

In this review we examine the potential role of nutritional approaches to reduce FS in PAD treatment. Given the established role of inflammation and OS in FS development, the MedDiet—rich in flavonoids and omega-3 fatty acids—may effectively mitigate FS and improve treatment outcomes in PAD patients through its anti-inflammatory and antioxidant properties.

The prevalence of PAD increases with age [62], and FS, which is closely associated with aging, is common in elderly populations [56]. Despite multiple available treatment options for PAD, challenges remain in patient selection and optimal treatment timing. Some patients may not fully benefit from these therapies due to factors such as frailty or comorbidities [127]. In PAD patients, FS exacerbates muscle atrophy, impairs physical function, and increases the likelihood of poor treatment outcomes, including inadequate responses to endovascular and surgical interventions [80]. Furthermore, FS has been identified as an independent risk factor for mortality and complications in PAD patients undergoing surgical or endovascular procedures [84].

Poor nutritional status is prevalent among frail patients awaiting vascular surgery and is associated with prolonged hospitalization, impaired functional recovery, and delayed wound healing [128]. Incorporating polyphenols and omega-3 fatty acids into clinical care protocols may improve muscle health, vascular function, and overall survival in frail PAD patients [129,130]. The flavonoid-rich and omega-3-abundant MedDiet is considered an effective option for improving patient health. By reducing chronic inflammation and OS—key contributors to vascular dysfunction and muscle degeneration in PAD—this dietary approach may be beneficial [10,15]. The current article examines the prominent role of flavonoids and omega-3 fatty acids in reducing FS and improving treatment outcomes in PAD patients.

Inflammatory and OS pathways, which contribute to PAD development and unfavorable clinical outcomes, are directly linked to frailty characterized by increased vulnerability to stressors and diminished physiological reserves [17]. This article specifically examines the biological pathways through which flavonoids and omega-3 fatty acids may reduce inflammatory conditions and OS. Although direct evidence linking some of these signaling pathways to FS or age-related diseases remains limited or insufficient, studies conducted in cellular and animal models or in the context of other aging-related processes suggest a biologically plausible connection. Based on this, we hypothesize that these pathways may similarly contribute to the reduction in FS. In the following sections, we will discuss each of these pathways individually.

### 5.1. Anti-Inflammatory Properties and Mechanisms of Flavonoids

#### 5.1.1. NF-κB Pathway

The Nuclear factor kappa (NF-κB) -light-chain-enhancer of activated B cells pathway plays a central role in regulating inflammation by promoting the expression of various cytokines, including TNF-α, IL-6, and IL-1β, as well as adhesion molecules like soluble vascular cell adhesion molecule-1 (sVCAM-1) and soluble intercellular adhesion molecule-1 (sICAM-1), which are involved in vascular inflammation [131]. CRP synthesis is primarily driven by inflammatory cytokines like IL-6 and TNF-α, which play key roles in orchestrating inflammatory responses and modulating immune function [132]. Activation of NF-κB also enhances the expression of pro-inflammatory factors like platelet cell adhesion molecule-1 (pCAM-1), which is linked to monocyte adhesion and endothelial dysfunction [133]. This pathway is crucial in the pathogenesis of diseases such as atherosclerosis and CVDs [134,135,136,137].

**Figure 1:**
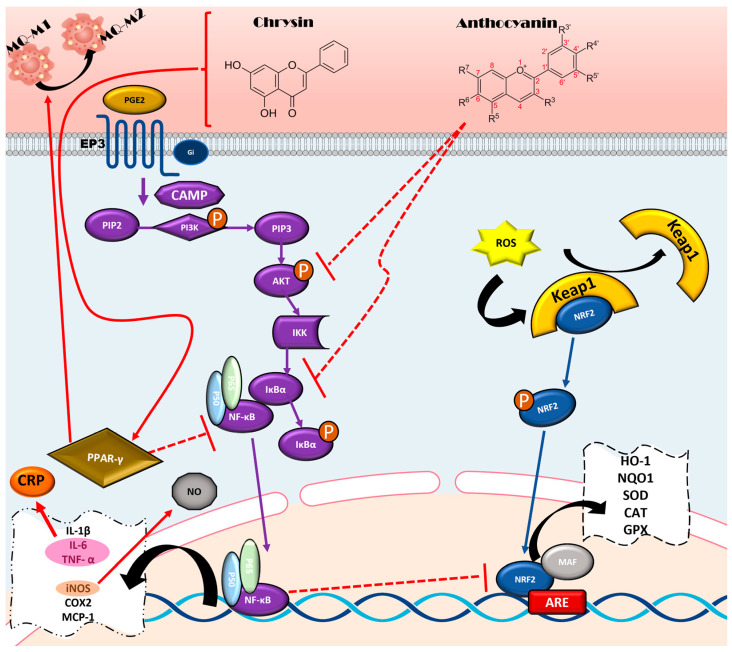
Signaling pathway of the anti-inflammatory mechanism of flavonoids: flavonoids like chrysin and anthocyanins exert anti-inflammatory effects by inhibiting the NF-κB signaling pathway, promoting macrophage polarization toward the M2 phenotype via PPARγ activation, and enhancing antioxidant gene expression through the Keap1/Nrf2 axis.

NF-κB usually kept in the cytoplasm by the inhibitor of nuclear factor kappa-B alpha (IkBa). When IkBa is phosphorylated, NF-κB is activated, leading to the expression of pro-inflammatory genes and cytokines such as TNF-α, IL-1β, IL-6, and Cyclooxygenase-2 (COX-2), which contribute to inflammation [138,139,140]. Several sources of *Anthocyanins*, a type of Flavonoids, have demonstrated potent inhibitory effects on NF-κB activation by stopping IκBα from being phosphorylated and degraded [133,141]. For instance, *Anthocyanins* in strawberries profoundly prevent the NF-κB p65 subunit from translocating, which in turn suppresses macrophage inflammatory responses [142,143]. Other flavonoids such as *Fisetin* have also been shown to regulate NF-κB signaling [144] (Figure 1).

#### 5.1.2. Regulation of Macrophage Polarization by PPARγ Pathway

Macrophages are central to metabolic disorders and their associated diseases. The polarization of macrophages into M1 and M2 subtypes plays a key role in modulating the inflammatory response. M1 macrophages typically contribute to pro-inflammatory effects, while M2 macrophages exhibit anti-inflammatory and tissue repair properties [145]. The balance between these subtypes is crucial for maintaining immune and inflammatory homeostasis, with macrophage polarization heavily influenced by the surrounding inflammatory microenvironment [146].

Peroxisome proliferator-activated receptor gamma (PPARγ) is a ligand-inducible transcription factor that regulates adipogenesis and modulates low-grade inflammation. It plays a crucial role in immune system regulation, influencing the differentiation and activation of immune cells, cytokine expression, and cell fate determination, thus maintaining immune balance [147]. PPARγ activation has been shown to promote the polarization of macrophages toward an anti-inflammatory M2 phenotype, helping to inhibit chronic inflammation and mitigate diseases associated with metabolic disorders [148]. PPARγ ligands, such as flavonoids like *chrysin*, have been demonstrated to exert significant anti-inflammatory effects by modulating macrophage polarization, providing a potential therapeutic strategy for treating inflammation [149].

Also, the anti-inflammatory effects of PPARγ agonists are mediated through repression of NF-κB target genes, indicating cross-talk between PPARγ and NF-κB signaling pathways. In models of high-fat diet-induced inflammation, PPARγ activation has been shown to inhibit NF-κB activation, supporting the role of PPARγ modulators in inflammation regulation [150] (Figure 1).

#### 5.1.3. IL-17

*Anthocyanidin*, a flavonoid commonly found in red berries, has been shown to reduce inflammation in various diseases, including asthma, diabetes, atherosclerosis, and cancer, primarily due to its anti-inflammatory properties [151,152,153,154,155]. This flavonoid’s effect on the IL-17A/IL-17RA signaling pathway is particularly noteworthy. The inhibition of this interaction by *Anthocyanidin* helps explain its anti-inflammatory activity, especially since IL-17A is a key cytokine involved in numerous chronic inflammatory conditions, such as rheumatoid arthritis, multiple sclerosis, experimental autoimmune encephalomyelitis (EAE), allergen-induced pulmonary inflammation, psoriasis, and cancer [156,157,158,159,160].

### 5.2. Antioxidant Properties and Mechanisms of Flavonoids

Excessive production of ROS does not just damage cells, it is also closely linked to aging process and is implicated in the development of age-related diseases such as cardiovascular conditions, neurodegenerative disorders in older adults [23]. ROS can damage mitochondrial DNA, disrupt organelle function, and increase the release of pro-inflammatory cytokines like TNF-α, which further accelerates cellular deterioration [161]. Overall, OS plays a major role in conditions such as metabolic syndrome, inflammatory skin disorders, and neurological diseases [141,162,163].

**Figure 2:**
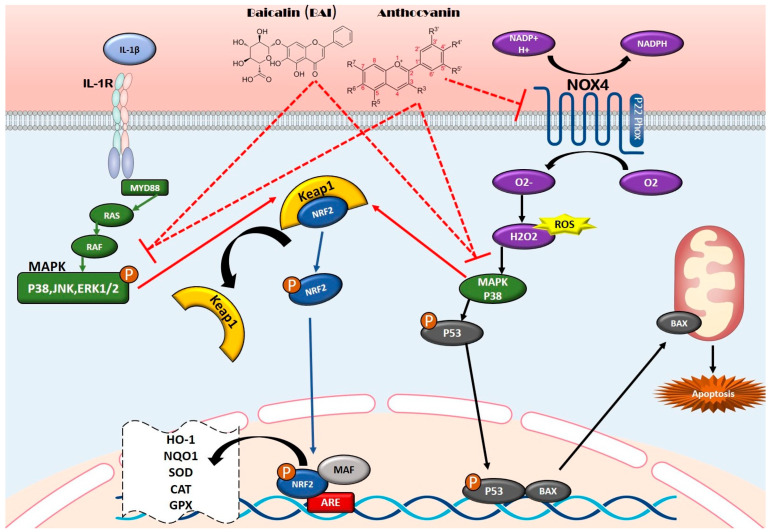
Signaling pathway of the Antioxidant mechanism of flavonoids: flavonoids, particularly anthocyanins, exhibit strong antioxidant properties by scavenging ROS and enhancing endogenous antioxidant enzymes such as GPx, CAT, and SOD. Their antioxidant effects are mediated through the Nrf2 signaling pathway, promoting cytoprotective enzyme expression while inhibiting MAPK signaling and NADPH oxidase (Nox4), ultimately reducing oxidative stress and inflammation. Arrows with curved ends indicate activation, while arrows with straight ends and dashed lines represent inhibition. The different colors of the arrows are used to distinguish between various signaling pathways.

Flavonoids, especially *Anthocyanins*, help counteract OS by scavenging ROS and boosting the activity of key antioxidant enzymes like glutathione peroxidase (GPx), catalase (CAT), and SOD. A central mechanism behind this antioxidant effect is the Nrf2 signaling pathway. When oxidative stress activates Nrf2, it moves into the cell nucleus and binds to a DNA region called the antioxidant response element (ARE), triggering the expression of protective enzymes like heme oxygenase-1 (HO-1), which help defend cells against ROS. Studies have shown that inhibiting the MAPK pathway enhances Nrf2 activity, leading to increased antioxidant enzyme production. On the flip side, activation of Erk1/2—a part of the MAPK pathway that can suppress Nrf2—worsens OS [164,165]. Some flavonoids, like *Baicalin*, appear to do both: activate the Nrf2 pathway and inhibit MAPK signaling, highlighting their dual role in reducing oxidative stress and controlling inflammation [166]. (Figure 2)

*Anthocyanins* in blueberries also enhance their antioxidant potential by downregulating NADPH oxidase 4 (Nox4), a key enzyme responsible for generating ROS [167] (Figure 2).

Omega-3 fatty acids have been shown to reduce oxidative stress and inflammation, both of which are key contributors to vascular fragility in patients with PAD [168].

### 5.3. Anti-Inflammatory Mechanisms of Omega-3 Fatty Acids

Extensive research highlights the regulatory role of omega-3 fatty acids in inflammation. Unlike omega-6 fatty acids, which often promote inflammatory responses, omega-3s exert anti-inflammatory effects through the downregulation of critical cytokines such as TNF-α, IL-6, and CRP biomarkers strongly associated with FS [169,170,171].

Deficiency in omega-3s, particularly in aging populations, has been linked to elevated IL-6 levels, a cytokine implicated in sarcopenia [169]. A meta-analysis by Custodero et al. [172] confirmed that omega-3 supplementation significantly reduces CRP and IL-6 levels in middle-aged and older adults. Similarly, a randomized controlled trial by Daboit et al. [173] demonstrated that supplementation with eicosapentaenoic acid (EPA) and docosahexaenoic acid (DHA) led to significant reductions in IL-6, IL-1β, and TNF-α, with more pronounced effects after 8 weeks of continuous use.

Furthermore, a study among Greek adults (aged 18–91 years) revealed that omega-3 intake decreased CRP levels [174]. Magee et al. [175] in a separate study, reported a notable increase in the IL-10/IL-6 ratio among participants who received omega-3 fatty acid supplementation compared to those given a placebo. This shift toward an anti-inflammatory profile suggests that omega-3 fatty acids may offer protective benefits by counteracting the harmful effects of key pro-inflammatory mediators such as IL-6 and TNF-α.

**Figure 3:**
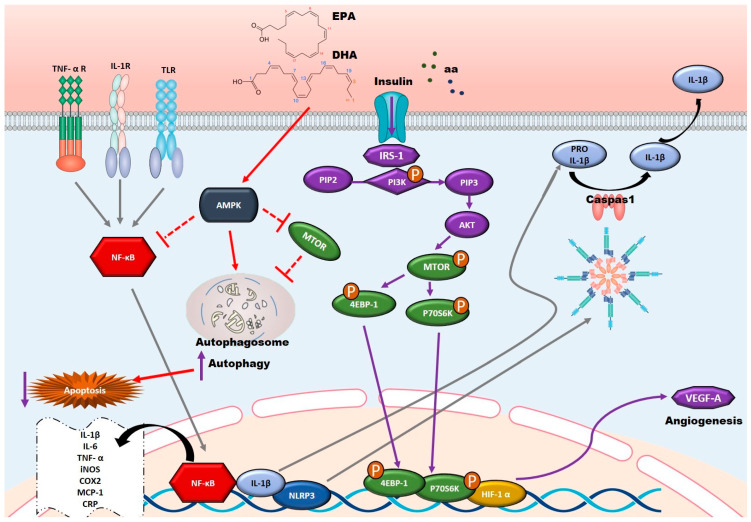
Signaling pathway of the anti-inflammatory mechanism of omega-3 fatty acids: omega-3 fatty acids exert anti-inflammatory effects by modulating AMPK and NF-κB pathways, reducing pro-inflammatory cytokines (TNF-α, IL-6, CRP), and enhancing IL-10 levels. They promote vascular repair via PI3K-mTOR signaling, improving endothelial function and reducing restenosis risk. Arrows with curved ends indicate activation, while arrows with straight ends and dashed lines represent inhibition. The different colors of the arrows are used to distinguish between various signaling pathways.

Omega-3 fatty acids also modulate major signaling pathways such as the NF-κB pathway and the NLRP3 inflammasome. A key mechanism includes activation of AMP-activated protein kinase (AMPK), which deacetylates and suppresses NF-κB activity, ultimately reducing inflammation [21,176,177]. Intake of alpha-linolenic acid (ALA), a plant-derived omega-3, has been shown to reduce pro-inflammatory mediators like TNF-α and COX-2, potentially helping to prevent age-related conditions such as diastolic dysfunction [129] (Figure 3).

Also, McGlory et al. [129] found that increased omega-3 intake, particularly EPA and DHA, significantly improved muscle mass and strength in older adults. These effects were also linked to enhanced immune function and reduced systemic inflammation.

### 5.4. Antioxidant Properties and Mechanisms of Omega-3 Fatty Acids

**Figure 4:**
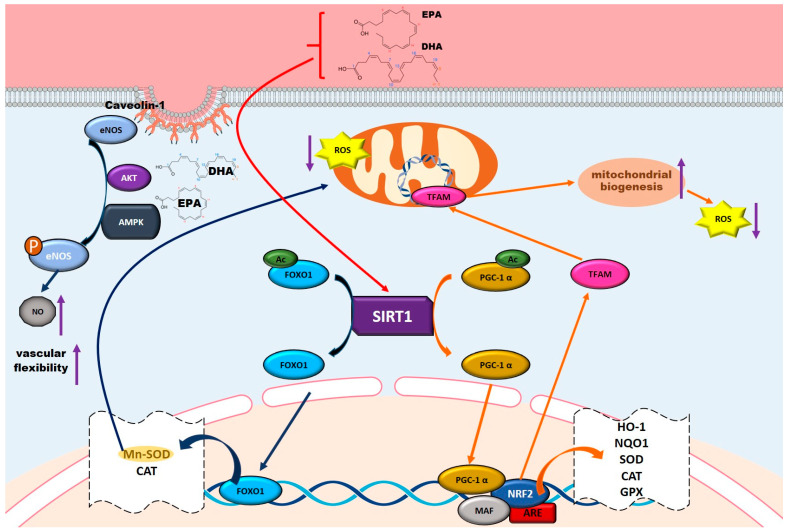
Signaling pathway of the antioxidant mechanism of omega-3 fatty acids: N-3 PUFAs improve endothelial function by increasing nitric oxide (NO) bioavailability and modulating antioxidant proteins like SIRT1 and FOXO, which enhance the expression of enzymes such as SOD. DHA boosts mitochondrial Mn-SOD activity, while omega-3s promote mitochondrial biogenesis via PGC-1α and NRF2 activation, reducing oxidative stress and telomere erosion. These mechanisms collectively enhance vascular flexibility. Arrows with curved ends indicate activation, while arrows with straight ends and dashed lines represent inhibition. The different colors of the arrows are used to distinguish between various signaling pathways.

Findings from an observational study suggest that increased OS reflected by elevated SOD activity and reduced total antioxidant capacity against ROS may be associated with telomere shortening, thereby contributing to biological aging and the progression of FS [26]. Omega-3 fatty acids promote mitochondrial biogenesis through the up-regulation and deacetylation of peroxisome proliferator-activated receptor gamma coactivator 1-alpha (PGC-1α), thereby augmenting mitochondrial functionality and enhancing cellular resilience to OS [178,179]. This mechanism is intricately associated with the activation of NRF2, which increases the expression of antioxidant enzymes such as glutathione peroxidase (GPx), catalase (CAT), and SOD. Collectively, these enzymes alleviate cellular oxidative stress and inhibit telomere erosion [178,179,180] (Figure 4).

Furthermore, these compounds modulate key proteins such as Sirtuin 1 (SIRT1) and forkhead box O (FOXO), which regulate the expression of antioxidant enzymes like SOD, providing cellular protection against oxidative damage [178,179,180,181] (Figure 4).

### 5.5. Vascular Protective Role of Omega-3 Fatty Acids

Alonso-Bouzón et al. [182] were the first to highlight the connection between endothelial dysfunction and frailty in older individuals. These findings provide additional support for the significant role of the vascular system in FS from the early stages of vascular disease.

The endothelium acts as a crucial interface between tissues and the circulating blood, functioning not only as a physical barrier but also as a regulator of vascular homeostasis. It inhibits the aggregation and adhesion of platelets and leukocytes and synthesizes several vasoactive substances, including nitric oxide (NO) and endothelin, which help maintain vascular tone and integrity [183]. Disruption in the balance between endothelial cell damage and repair mechanisms contributes to structural changes in the vascular wall, a process known as vascular remodeling, and reduces the bioavailability of NO [183,184].

Emerging evidence indicates that endothelial dysfunction serves not only as an early predictive marker of FS (prior to clinical manifestation) [185], but also pathophysiologically confirms the central role of vascular impairment in FS development. This association becomes clinically significant from the earliest stages of vascular dysfunction when only endothelial dysfunction is detectable [182].

Omega-3 fatty acids play a key role in maintaining vascular health and preventing the progression of atherosclerosis by improving endothelial function, reducing platelet aggregation, and enhancing membrane fluidity [186].

#### 5.5.1. Activating the PI3K-AKT-mTOR Pathway for Vascular Repair

The regulation of angiogenesis essential for vascular repair relies on tightly controlled pathways, including vascular endothelial growth factor A (VEGF-A) signaling. The phosphoinositide 3-kinase—protein kinase B (AKT)—mammalian target of rapamycin (PI3K-AKT-mTOR) pathway plays a central role in this process, and omega-3 supplementation has been shown in animal studies to reduce pathological angiogenesis, indicating therapeutic potential in vascular healing [162,187].

Omega-3 polyunsaturated fatty acids (n-3 PUFAs) enhance mTOR activation by improving insulin sensitivity and increasing amino acid availability. This leads to downstream signaling through p70S6K and 4E-BP1, thereby promoting cellular growth and repair [188]. Within the vascular endothelium, mTOR signaling regulates endothelial cell proliferation, migration, and survival key processes for optimal arterial repair, particularly following stent placement [189].

Moreover, omega-3s increase nitric oxide bioavailability, which improves vascular tone and reduces the risk of restenosis [190]. These fatty acids also regulate autophagy pathways, helping to suppress excessive smooth muscle cell proliferation, a major cause of complications after stent implantation [191].

In addition, omega-3 supplementation boosts mitochondrial activity, reducing cellular stress and enhancing the processes involved in endothelial homeostasis and vascular regeneration [192] (Figure 3)**.**

#### 5.5.2. Enhancing NO Production and Endothelial Function

PUFAs exert their antioxidant effects primarily by enhancing endothelial function. These fatty acids increase the bioavailability of NO, a crucial factor for maintaining endothelial integrity and mitigating oxidative stress that contributes to arterial stiffness [191,192,193]. Research indicates that omega-3 PUFAs facilitate NO synthesis by altering caveolae composition, thereby enhancing endothelial performance and reinforcing antioxidant activities [194,195,196,197,198].

### 5.6. The Bioavailability of Anthocyanins and Omega-3

Anthocyanins, known as antioxidant-rich plant pigments, exhibit extremely low bioavailability—less than 1% and are therefore swiftly metabolized into phenolic compounds such as protocatechuic acid (PCA) and vanillic acid (VA) [199,200]. These resulting metabolites, which achieve higher concentrations in plasma and remain active for up to 48 h, are considered key contributors to improved endothelial function and vascular protection [201,202,203]. Clinical evidence indicates that the intake of anthocyanin-rich fruits, particularly blueberries and strawberries, is linked to enhanced flow-mediated dilation (FMD) and decreased markers of oxidative stress [202,204,205]. Nevertheless, the poor bioavailability of anthocyanins continues to limit their clinical efficacy, prompting ongoing research into strategies to boost their absorption and stability. Among these, encapsulation techniques such as lipid-based nanoparticles and water-in-oil-in-water (W/O/W) nanoemulsions have shown promise, with the potential to improve anthocyanin stability by up to 94.6% [206,207]. Moreover, structural modifications like acylation have been reported to enhance resistance against photodegradation [208]. Co-administration with compounds like proteins or simple sugars (e.g., sucrose) may also extend the thermal stability of anthocyanins, increasing their half-life by four to fivefold [209,210]. Importantly, evidence supports the use of metabolite mixtures rather than isolated anthocyanins, emphasizing the complexity of maximizing their biological effects in vivo [211,212,213].

In contrast, the bioavailability of omega-3 fatty acids is affected by various factors, including the dietary source (e.g., fish oil vs. plant-based oils), chemical structure (triglycerides vs. ethyl esters), and individual metabolic conditions. Notably, omega-3 from fish oil is more efficiently absorbed and produces greater improvements in lipid profiles compared to plant-derived options such as flaxseed oil [214,215].

To provide more objective evidence from validated clinical trials, several key studies have highlighted the protective effects of natural compounds such as EPA and epicatechin on cardiovascular and muscle-related outcomes.

The REDUCE-IT trial demonstrated that daily supplementation with 4 g of icosapent ethyl (IPE)—a purified and stable form of EPA—in patients with elevated triglyceride levels and high cardiovascular risk resulted in a 25% reduction in major adverse cardiovascular events, including cardiovascular death, myocardial infarction, stroke, and the need for revascularization [216].

Similarly, the COCOA-PAD randomized clinical trial showed that daily consumption of a cocoa flavonoid-rich beverage containing 75 mg of epicatechin over a six-month period significantly improved walking capacity in patients with peripheral artery disease (PAD) [217,218]. Muscle biopsy analyses revealed that this intervention led to the upregulation of key Nrf2 target genes, such as HO-1 and NQO1, a reduction in markers of myopathy, and increased expression of UQCRC2—a critical component of the mitochondrial respiratory chain—indicating enhanced mitochondrial function [217]. The proposed mechanisms include increased Nrf2 phosphorylation, decreased production of reactive oxygen species (ROS), and restoration of mitochondrial function under PAD-induced oxidative stress. These effects were also replicated in vitro using patient-derived serum [217]. Overall, epicatechin—via Nrf2 activation and mitochondrial enhancement—emerges as a promising natural therapeutic agent for managing muscle-related complications in PAD, although larger clinical trials are needed to confirm its efficacy [217].

Further supporting the cardiovascular benefits of omega-3 fatty acids, a large cohort study reported that co-administration of omega-3s and statins significantly reduced the risk of all-cause mortality, adverse outcomes following endovascular procedures, and limb amputation in a dose-dependent manner in hemodialysis patients with dyslipidemia [219]. In addition, a comprehensive meta-analysis of 40 studies involving 135,267 participants demonstrated a strong and dose-dependent association between omega-3 intake and a reduced risk of cardiovascular disease and myocardial infarction [220].

These clinical findings underscore the therapeutic potential of EPA and epicatechin in reducing cardiovascular risk and improving functional outcomes in high-risk populations, particularly those with PAD, through mechanisms involving mitochondrial support, anti-inflammatory effects, and vascular protection.

Emerging evidence indicates that dietary flavonoids can help alleviate clinical complications linked to both frailty syndrome and peripheral arterial disease (PAD). A prominent issue in individuals affected by PAD and frailty is impaired vascular function, which disrupts endothelial integrity and reduces blood flow efficiency [202,205]. Interestingly, the intake of flavonoid-rich foods—such as blueberries—has been shown to significantly enhance flow-mediated dilation (FMD) by 1.50% and improve the reactive hyperemia index (RHI) by 0.26, reflecting better endothelial responsiveness and vascular performance [202,205]. These benefits are thought to stem from increased nitric oxide (NO) bioavailability and decreased oxidative stress—two essential mechanisms that support vascular health and may counteract complications associated with PAD [221,222].

Another key symptom of PAD is intermittent claudication, which may also be mitigated through flavonoid supplementation [223,224]. Several small-scale clinical trials have underscored the beneficial effects of cocoa-derived flavonoids in enhancing functional capacity among PAD patients. For example, Loffredo et al. [225] reported that, within two hours of consuming 40 g of dark chocolate containing more than 85% cocoa, 20 patients aged 60–70 years (14 men and 6 women) experienced notable improvements in walking distance, elevated levels of NOx, and a reduction in isoprostanes—markers of oxidative stress—compared to those who consumed milk chocolate (≤35% cocoa). Likewise, a six-month study by McDermott et al. [226], involving 44 older men aged 70–80 years, found that daily consumption of a synthetic cocoa beverage with 15 g of cocoa significantly increased walking distance relative to a placebo group. Collectively, these findings underscore the therapeutic promise of flavonoid-enriched dietary interventions—especially those derived from blueberries and cocoa—in improving endothelial function, reducing oxidative stress, and enhancing mobility and physical endurance in patients suffering from PAD and frailty syndrome.

Our findings align with existing evidence suggesting that healthy diets, particularly the Mediterranean diet rich in these compounds, may positively impact frailty risk reduction in elderly populations [227]. These results support the hypothesis that proper nutrition may be an important factor in preventing FS progression in PAD patients.

However, it should be noted that most existing studies have focused on general populations or patients with other chronic conditions, with limited research specifically targeting PAD patients. Furthermore, there is a notable lack of prominent studies examining the effects of nutritional interventions on frailty reduction and treatment outcome improvement in PAD.

While current evidence suggests that dietary modifications can reduce inflammation and improve clinical outcomes, insufficient data exist to conclusively confirm this hypothesis. Therefore, further research is necessary to better understand this relationship and its impact on PAD patient prognosis [227].

In conclusion, flavonoids and omega-3 fatty acids may help reduce FS and improve treatment efficacy in PAD patients through their anti-inflammatory and antioxidant properties. However, additional studies are required to confirm this hypothesis and evaluate their clinical effectiveness.

## 6. Conclusions

Despite multiple therapeutic options for PAD, challenges persist, including appropriate patient selection, optimal timing of interventions, and incomplete treatment responses particularly in patients with FS or comorbidities. In this context, preoperative frailty assessment has gained increasing importance for informed decision-making, especially in surgical and endovascular interventions. The ERAS (Enhanced Recovery After Surgery) society also recommends comprehensive geriatric assessment and personalized interventions for high-risk frail patients.

Current evidence underscores the high potential of flavonoid and omega-3-rich diets, such as the MedDiet, as non-invasive, cost-effective, and accessible nutritional interventions to reduce frailty, preserve muscle function, and promote vascular repair. However, well-designed longitudinal clinical trials are needed to confirm the clinical efficacy of these compounds, determine optimal dosages, and identify synergistic effects in specific PAD populations.

Ultimately, future research should focus on patient-reported quality of life in frail patients before and after different treatments, as well as on the clinical and cost-effectiveness of preoperative frailty screening and rehabilitation strategies. This approach could revolutionize the nutritional and therapeutic management of frail PAD patients, offering a comprehensive pathway to improving cardiovascular health in the aging population.

## Data Availability

We used PubMed, SCOPUS, and ScienceDirect databases to screen articles for this review. We did not report any data.

## Abbreviations

The following abbreviations are used in this manuscript:

PAD	Peripheral artery disease
MedDiet	Mediterranean diet
OS	Oxidative stress (OS)
RONS	reactive oxygen and nitrogen species
hs-CRP	High-sensitivity C reactive protein
GSSG	oxidized glutathione
MDA	Malondialdehyde
4-HNE	4-hydroxy-2-nonenal
SOD	superoxide dismutase
IL-6	interleukin-6
ROS	reactive oxygen species
TNF-α	tumor necrosis factor alpha
IL-1β	interleukin-1 beta
IL-18	interleukin-18
IL-8	interleukin-8
CXCL10	chemokine (C-X-C) motif ligand 10
CVD	cardiovascular disease
T2D	type 2 diabetes
MetS	metabolic syndrome
NHS	Nurses’ Health Study
CHD	coronary heart disease
HPFS	Health Professional Follow-up Study
MI	myocardial infarction
AHA	American Heart Association
ESC	European Society of Cardiology
IC	Intermittent Claudication
GFI	Groningen Frailty Indicator
mEFT	modified Essential Frailty Toolset
CLTI	Chronic limb-threatening ischemia
mFI	Modified Frailty Index
ABI	Ankle-Brachial Index
NF-κB	Nuclear Factor kappa-light-chain-enhancer of activated B cells
sVCAM-1	Soluble Vascular Cell Adhesion Molecule-1
sICAM-1	Soluble Intercellular Adhesion Molecule-1
pCAM-1	Platelet Cell Adhesion Molecule-1
COX-2	Cyclooxygenase-2
ERK	Extracellular Signal-Regulated Kinase
MAPK	Mitogen-Activated Protein Kinase
SAPK	Stress-Activated Protein Kinase
JNK	c-Jun N-terminal Kinase
PPARγ	Peroxisome Proliferator-Activated Receptor Gamma
GPx	Glutathione Peroxidase
CAT	Catalase
ARE	antioxidant response element
HO-1	Heme Oxygenase-1
Nox4	NADPH oxidase 4
AMPK	AMP-Activated Protein Kinase
ALA	alpha-linolenic acid
EPA	Eicosapentaenoic Acid
DHA	Docosahexaenoic Acid
AA	arachidonic acid
PI3K-AKT-Mtor	Phosphoinositide 3-Kinase-Protein Kinase B (AKT)-Mammalian Target of Rapamycin
VEGF-A	Vascular Endothelial Growth Factor A
n-3 PUFAs	Omega-3 polyunsaturated fatty acids
SIRT1	Sirtuin 1
FOXO	Forkhead Box O
PGC-1α	Peroxisome Proliferator-Activated Receptor Gamma Coactivator 1-alpha
NO	nitric oxide
